# Synergistic effects of sesame oil, extra virgin olive oil, psyllium extract, and dandelion extract on cholesterol gallstone dissolution: An in vitro comparative study against Rowachol^®^

**DOI:** 10.1371/journal.pone.0334496

**Published:** 2025-10-14

**Authors:** Raghad Serri, Nada Dehneh, Mohammad Ghannam, Mohamad Radwan Sirri

**Affiliations:** 1 Department of Biochemistry and Microbiology, Faculty of Pharmacy, Arab International University, Ghabagheb, Daraa, Syria; 2 Department of Pharmacognosy and Pharmacology, Faculty of Pharmacy, Arab International University, Ghabagheb, Daraa, Syria; 3 Department of Orthodontics, University of Damascus Dental School, Damascus, Syria; Sapporo Medical University, JAPAN

## Abstract

**Background:**

Cholesterol gallstones represent a significant global health burden. Current treatments, including surgery and oral dissolution agents, are often invasive or limited by side effects and variable efficacy. This study investigated the in vitro gallstone-dissolving efficacy of a natural combination—sesame oil (SO), extra virgin olive oil (EVOO), psyllium extract (PE), and dandelion extract (DE)—compared to the pharmaceutical agent Rowachol®.

**Methods:**

In a randomized in vitro study, seventy cholesterol-dominant human gallstones were assigned to seven groups receiving either individual agents, multi-component combinations, or Rowachol® (control). Two prespecified endpoints were assessed under standardized simulated bile conditions at 48 h (T1), 96 h (T2), and 144 h (T3): dissolution rate (DR, %; weight loss) and cumulative cholesterol release (mg). Statistical analyses compared groups across time and explored potential multi-component interactions.

**Results:**

The four-component regimen (G6: PE + DE+SO+EVOO) showed the greatest efficacy at T3, achieving DR = 92.57% ± 4.2 and cholesterol release = 114.48 ± 4.2 mg, significantly exceeding Rowachol® (39.71% ± 1.9; 42.57 ± 1.9 mg; p < 0.001) and all other groups. Effects were time-dependent, with progressive separation from T1 to T3. Key bioactive compounds—oleic acid, taraxacin, arabinoxylan, and linoleic acid—showed strong positive correlations with dissolution outcomes (r = +0.76 to +0.94). A regression model identified these compounds as primary efficacy predictors, accounting for 94% of the observed variance (adjusted R² = 0.94).

**Conclusions:**

Under short-term, controlled in vitro conditions, the SO+EVOO+PE + DE combination achieved a ~ 2.3-fold higher dissolution rate than Rowachol® at 144 h. These findings constitute mechanistic, hypothesis-generating evidence that clarifies how dissolution may be enhanced ex vivo. Confirmation in well-designed in-vivo models—followed by clinical studies to evaluate safety, dosing, and effectiveness—is required before any patient-care application.

## 1. Introduction

Gallbladder gallstones are a major global health issue, affecting about 6.1% of people [1]. Their prevalence varies by region and economic status, with higher rates in South America (11.2%) and low-income countries (8.9%) compared to Asia (5.1%) and high-income nations (4%) [1]. Incidence rises with age, affecting 13% of adults under 70 [2], and shows a gender imbalance, occurring in 7.6% of females versus 5.4% of males [3]. These variations are attributed to hormonal factors and dietary habits, particularly high-fat diets [4].

Gallstones are classified into three main types: cholesterol, pigment, and mixed stones [5]. Cholesterol stones account for 80% of cases, contain up to 70% cholesterol, and are strongly associated with obesity [5]. Pigment stones contain 30% or less cholesterol and are further categorized into brown stones (linked to bile duct infections) and black stones (associated with hemolytic anemia or liver cirrhosis) [5]. Mixed stones contain 30% to 70% cholesterol [5]. Chemical analysis guides treatment choices. It helps decide between dissolution therapy and surgery. It also aids in developing preventive strategies by revealing links between stone composition, environmental factors, and health conditions [6].

Current gallstone treatments depend on stone type and location. Cholecystectomy (gallbladder removal) is a definitive solution but carries risks such as infection and bleeding [7]. Endoscopic retrograde cholangiopancreatography (ERCP) is effective for extracting bile duct stones but may cause ductal injury [8]. Extracorporeal shock wave lithotripsy (ESWL) fragments stones but can lead to temporary bile duct obstruction [7]. Chemical medications like bile acids dissolve small cholesterol stones over months but may cause gastrointestinal disturbances and hepatotoxicity [9].

Unlike conventional treatments, herbal remedies are widely used with minimal side effects (mainly mild digestive issues) [10]. In Morocco, 78.1% of patients use traditional herbal blends derived from 35 plant species, preserved through generations [11]. However, their efficacy and safety require further validation [10].

Among natural remedies, Sesame Oil (*Sesamum indicum*, Pedaliaceae), Extra Virgin Olive Oil (EVOO) (*Olea europaea*, Oleaceae), Psyllium Extract (*Plantago ovata*, Plantaginaceae), and Dandelion Extract (*Taraxacum officinale*, Asteraceae) have been traditionally used for gallstone treatment [12].

Sesame oil exhibits protective effects against gallstones through antioxidants like sesamin and sesamol, which reduce oxidative stress [13]. EVOO’s polyphenols, such as hydroxytyrosol and oleuropein, enhance bile secretion and prevent cholesterol crystallization, promoting stone dissolution [12,14,15]. Psyllium’s soluble fiber binds bile salts, reducing biliary cholesterol levels [16,17], whereas dandelion contains polyphenols and flavonoids that support liver function, enhance bile flow, and facilitate detoxification through diuretic properties [18–20]. S1 Table provides a detailed summary of these substances, their active components, and their mechanisms in gallstone prevention and treatment.

Recent in vitro studies highlight natural alternatives for gallstone dissolution. **Chekroune and Benamara** [21] revealed complete gallstone dissolution using an olive oil-lemon juice emulsion, likely due to synergistic actions between lipids and acidic compounds. **Sulaiman [22]** identified that a 3 mg/mL barley water extract effectively degraded cholesterol and mixed gallstones. Fourier-Transform Infrared Spectroscopy (FTIR) confirmed this effect, revealing a 31.8% reduction in carbon networks within the samples, indicative of gallstone disintegration.

**Tiwari and Sah** [23] achieved complete dissolution of cholesterol gallstones using 2 mg/mL apricot extract, releasing 377.3 mg/dL of cholesterol. **Arrout et al.** [24] demonstrated that Moroccan *Citrus sinensis* essential oil outperformed methyl tert-butyl ether (MTBE), dissolving 95.78% of cholesterol gallstones within 24 hours. **B.K et al.** [25] emphasized the effectiveness of *Berberis asiatica* against mixed cholesterol gallstones and *Taraxacum officinale* against black pigment gallstones, while Ayurvedic formulations such as Cystone®, Gokshuradi, and Calcury showed limited efficacy. Most recently, **Nodehi et al.** [26] reported that *Cichorium intybus* (chicory) and *Artemisia absinthium* (wormwood) extracts (500 mg/mL) degraded 75% of triglycerides and 63% of cholesterol in gallstones, significantly outperforming controls (P < 0.001 and P < 0.01, respectively).

Despite these advances, no comprehensive study has compared the effects of sesame oil, extra virgin olive oil, psyllium, and dandelion extracts on gallstones, leaving a critical gap in understanding their potential as natural therapeutic agents. This study aims to assess their synergistic effects on gallstone dissolution in vitro and identify the most effective bioactive compound.

## 2. Materials and methods

### 2.1. Settings and study design

This prospective study was conducted from December 2023 to August 2024 at the Biochemistry Department, Faculty of Pharmacy, Arab International University (AIU), Damascus, Syria, with ethical approval (Project No. 618–11GR, Protocol No. 1358).

### 2.2. Sample size assessment

**Fig 1:**
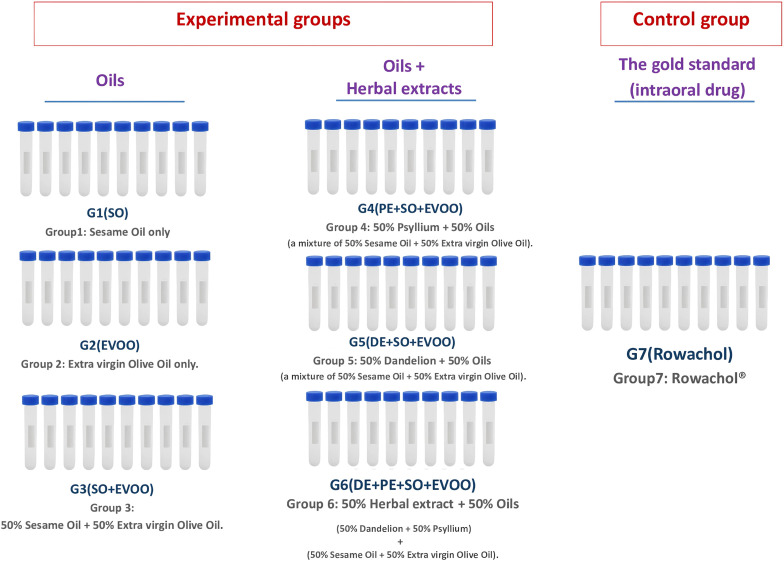
Study groups and interventions. Schematic of sample accrual, screening, and random allocation of cholesterol-dominant human gallstones into seven groups (n = 10 stones/group): G1 _(SO)_, G2 _(EVOO)_, G3 _(SO+EVOO)_, G4 _(PE+SO+EVOO)_, G5 _(DE+SO+EVOO)_, G6 _(PE+DE+SO+EVOO)_, and G7 _(Rowachol®)_. Stones were incubated under standardized simulated bile conditions and evaluated at T0 (baseline, 0 h), T1 (48 h), T2 (96 h), and T3 (144 h). Primary outcomes were dissolution rate (DR, %) and cumulative cholesterol release (mg). Abbreviations: **SO**, sesame oil; **EVOO**, extra virgin olive oil; **PE**, psyllium extract; **DE**, dandelion extract. **Source**: Created by the authors.

G*Power 3.1.7 (Universität Düsseldorf, Germany) was used to calculate the sample size. Based on a previous study [23], an effect size of 0.6 was assumed. A one-way ANOVA was used to compare the seven groups, with a significance level (α) of 0.05 and a power (1-β) of 0.95. The analysis determined that a minimum of 10 gallstones per group (70 total) was needed to detect a clinically meaningful difference in dissolution rates**. Fig 1** presents the study groups and their respective materials.

### 2.3. Participants and gallstone collection

This study **included** 55 patients (30 males, 25 females; mean age: 44 ± 1.15 years, range: 40–50 years; BMI: 20–30 kg/m²) with cholesterol gallstones requiring surgical removal. **Exclusion** criteria included: (1) chronic systemic diseases—such as cirrhosis, chronic kidney disease stage ≥ 3, congestive heart failure NYHA III–IV, and uncontrolled diabetes mellitus (HbA1c > 8.5%)—; (2) acute inflammatory or infectious conditions—such as acute cholecystitis, cholangitis, pancreatitis, or any febrile illness within the preceding 14 days—as well as hepatitis B/C; (3) pregnancy; (4) malignancy.

Gallstones were selected based on strict **criteria**: > 70% cholesterol composition (confirmed via FTIR), size 10–13 mm (measured with a digital caliper), weight 120–150 mg (using a sealed electronic balance), and semi-circular shape (visually verified). A total of 175 gallstones were collected from five private hospitals in Damascus, Syria (1 February–30 May 2024): Al-Mahaini Modern (10 patients/32 stones), Dar Al-Shifa (13 patients/43 stones), Al-Shami (14 patients/48 stones), Al-Salam Specialized (10 patients/27 stones), and Al-Kindi (8 patients/25 stones).

**Fig 2:**
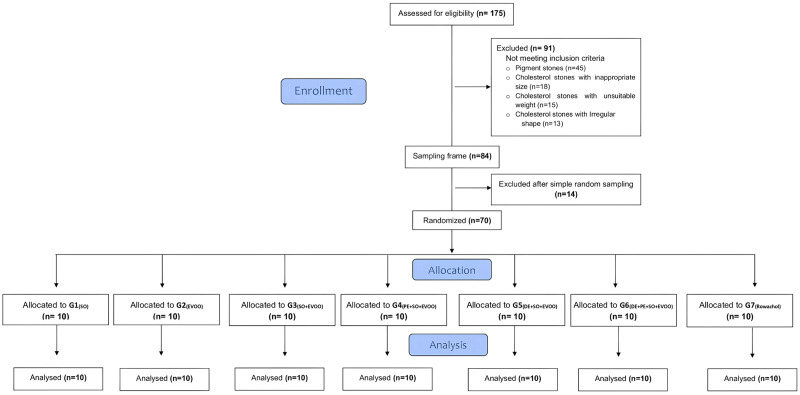
Flowchart of gallstone selection and allocation. Flow diagram showing the number of stones collected, exclusions per predefined criteria, and final allocation to the seven groups (n = 10 each). All measurements were performed on de-identified specimens from adult donors. **Source**: Created by the authors.

**Fig 3:**
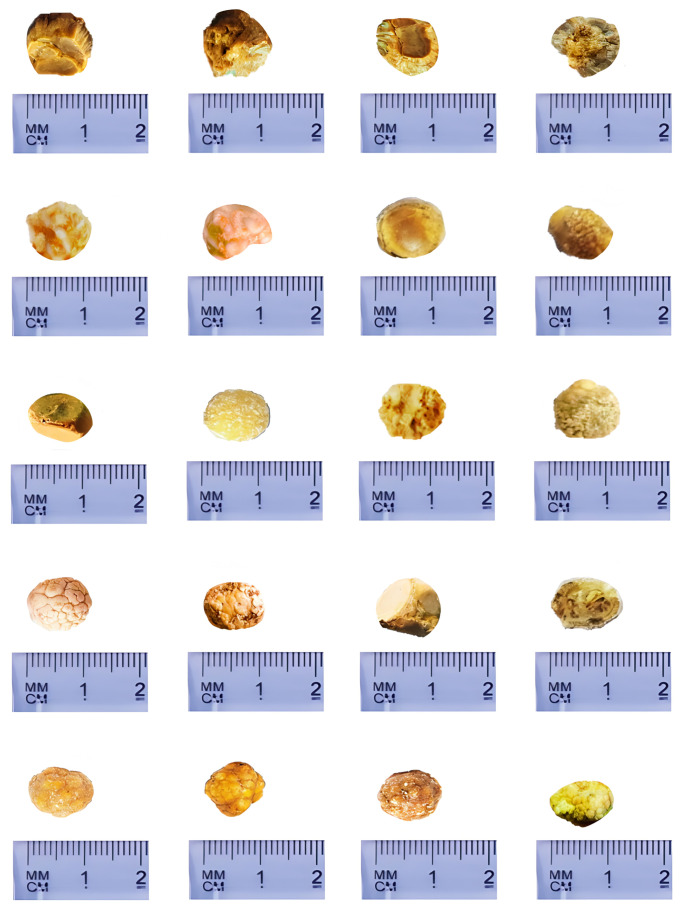
Representative Sample of Cholesterol Gallstones Used in the Study. Representative macro-images of included stones at T0 showing typical morphology and surface features prior to incubation. A scale bar is provided for size reference. Stones were cholesterol-dominant per material characterization described in Methods. **Source**: Created by the authors.

FTIR analysis identified 130 cholesterol-dominant stones, with 84 meeting inclusion criteria. After washing with distilled water, drying at 36°C for 5 hours, and reweighing, 70 gallstones were randomly selected for final analysis. The recruitment and selection process is summarized in **Fig 2**, and **Fig 3** displays representative gallstone samples from the study.

### Informed consent process

Prior to gallstone collection, all participants provided written informed consent after receiving a detailed explanation of the study’s purpose, procedures, and potential use of their gallstones for research. The consent form explicitly stated that the gallstones would be used in vitro experiments aimed at evaluating natural dissolution agents, and assured participants of the confidentiality and anonymity of their data. Patients were informed that their participation was voluntary and that they could withdraw at any time without affecting their medical care. All consent forms were stored securely, and only de-identified gallstone samples were used in the experiments to protect patient privacy.

### 2.4. Plant materials

Nutrient oils (sesame and EVOO) were extracted using protocols adhering to international standards (detailed in S2 Table), including solvent types, extraction durations, and storage conditions. Similarly, plant extracts (Psyllium seeds, dandelion) were obtained through sequential methods, as specified in S2 Table. Chemical compounds in oils were analyzed via a Gas Chromatography-Mass Spectrometry device (GC-MS, Shimadzu GCMS-QP2010 Ultra, Japan), with Kovats Index calculated for volatile compounds (fatty acids, phenolics). Non-volatile compounds (fibers, polyphenols, flavonoids) in plant extracts were identified using a High-Performance Liquid Chromatography device (HPLC, Agilent 1260 Infinity II, USA). Full details are provided in the S3 Table.

Rowachol® (A. Nattermann & Cie. GmbH, Germany), an FDA-approved gallstone dissolution drug, is prescribed as 1–2 capsules three times daily after meals. For experiments, its active terpenes were prepared at 100 mg/test tube (extraction method described in S4 Table).

Commercially sourced human bile juice (BioIVT, USA; HBC-GS-350ML) was used in all dissolution trials. Bile was stored at –80°C and thawed at 4°C for 12 hours pre-use. Physicochemical properties, storage protocols, and compliance details are in S5 Table.

### 2.5. Dissolution methods

GS dissolution experiments were performed at four intervals (T0: baseline, T1: 48h, T2: 96h, T3: 144h) using 70 round-bottom screw-cap tubes (15 mL), divided into 7 groups (6 experimental, 1 control). Each tube contains one gallstone immersed in 10 mL of dissolving fluid (BioIVT with 100 mg/mL dissolving agent) (see S6 Table). The entire bile-based solvent was replaced every 48 h to keep the medium below its critical micellar saturation point for cholesterol, thereby preserving the concentration gradient that drives dissolution. Routine renewal also prevents the accumulation of bilirubin and calcium ions, which can precipitate as calcium-bilirubinate/carbonate layers and insulate the stone surface. Released cholesterol was quantified by measuring total cholesterol in the replaced solvent (Cholestech LX-20 autoanalyzer, Beckman Coulter, USA) and subtracting the baseline bile cholesterol (4–6 mM). Gallstones were washed, dried, and weighed using a calibrated Ohaus Adventurer Pro AV-2102 balance (Ohaus Corporation, USA). The dissolution rate (DR%) was calculated as:


DR% = ((W_initial − W_final) / W_initial) × 100.

**DR%**: is the Dissolution Rate of the calculation, **W**_**initial**_: is the GS weight before incubation, and **W**_**final**_: is the GS weight after incubation.

All measurements (cholesterol and weight) were performed in triplicate, with arithmetic means ± standard deviations reported.

### 2.6. Randomization, blinding, validity, and reliability

Seventy cholesterol gallstones were divided into seven groups via computer-generated randomization. An independent researcher concealed group assignments in sealed opaque envelopes until experiments began. To maintain blinding, researcher R.S. prepared solutions, tubes, gallstones, and refreshed solvents but was excluded from subsequent measurements. Three blinded biochemistry specialists measured cholesterol levels and gallstone weights in triplicate. R.S. calibrated instruments daily, and all procedures occurred between 8 and 11 AM. Statistical analyses were conducted by blinded biostatistician M.Z.

### 2.7. Statistical analysis

Statistical analysis was performed using SPSS v26 (IBM Corp., USA). **Baseline** gallstone characteristics (weight, cholesterol) and dissolution parameters were summarized as mean ± SD. Normality was confirmed via Shapiro-Wilk tests. **Primary analyses**: one-way ANOVA (baseline weight homogeneity at T0), repeated measures ANOVA/linear mixed models (time [T0–T3], group [G1–G7], and interaction effects on DR% and cholesterol release). Post-hoc tests included Tukey’s HSD (all groups) and Dunnett’s test (experimental vs. control). **Secondary analyses**: Bonferroni-adjusted paired t-tests (within-group trends), Pearson correlations (chemical composition vs. outcomes), and multiple regression (key predictors). Reliability was assessed via intraclass correlation coefficients (ICC) for triplicate measurements.

## 3. Results

### 3.1. Baseline characteristics of patients and gallstones

The study included 55 patients (54.54% men, 45.45% women) with a mean age of 44 ± 1.15 years. Baseline characteristics of GSs (N = 70) revealed variations in weight (121.24 ± 1.8 mg to 148.29 ± 2.9 mg), size (10.2 ± 0.5 mm to 13.5 ± 1.0 mm), and chemical composition (cholesterol: 71.0 ± 2.5% to 77.3 ± 2.1%; bilirubin: 15.2 ± 1.0% to 32.8 ± 2.0%) across groups (Table 1).

**Table 1:** Initial Characteristics of the patients and their GSs (Baseline Measurements at T0).

Characteristics of the patients			Characteristics of the GSs				
Variable	Mean	SD	Group (N = 70)	Weight (mg) (T0) (Mean ± SD)	Size (mm) (T0) (Mean ± SD)	Chemical Compositions	
						Cholesterol (%) (T0) (Mean ± SD)	Bilirubin (%) (T0) (Mean ± SD)
Age (y)	44	1.15	G1 _(SO)_	121.24 ± 1.8	10.2 ± 0.5	77.3 ± 2.1	20.1 ± 1.2
			G2 _(EVOO)_	134.37 ± 2.3	10.1 ± 0.6	75.5 ± 2.3	22.4 ± 1.5
			G3 _(SO+EVOO)_	148.29 ± 2.9	10.5 ± 0.7	71.0 ± 2.5	25.8 ± 1.8
Gender (Men (%)/Women (%))	30 (54.54%)/25 (45.45%)		G4 _(PE+SO+EVOO)_	126.31 ± 2.1	11.3 ± 0.8	76.2 ± 2.0	28.5 ± 1.7
			G5 _(DE+SO+EVOO)_	139.07 ± 2.5	12.4 ± 0.9	73.7 ± 1.9	30.1 ± 1.9
			G6 _(PE + DE+SO+EVOO)_	135.16 ± 2.2	13.5 ± 1.0	74.4 ± 1.8	32.8 ± 2.0
			G7 _(Rowachol)_	141.09 ± 2.7	12.5 ± 0.4	72.8 ± 2.6	15.2 ± 1.0

GSs: gallstones; SD: Standard Deviation; G: Group; N: number of gallstones; y: year; SO: Sesame Oil; EVOO: Extra Virgin Olive Oil; PE: Psyllium Extract; DE: Dandelion Extract; T0: Baseline measurement; mg: milligrams; mm: millimeter.

### 3.2. Temporal Evolution of Dissolution Rates and Cholesterol Release

**Fig 4:**
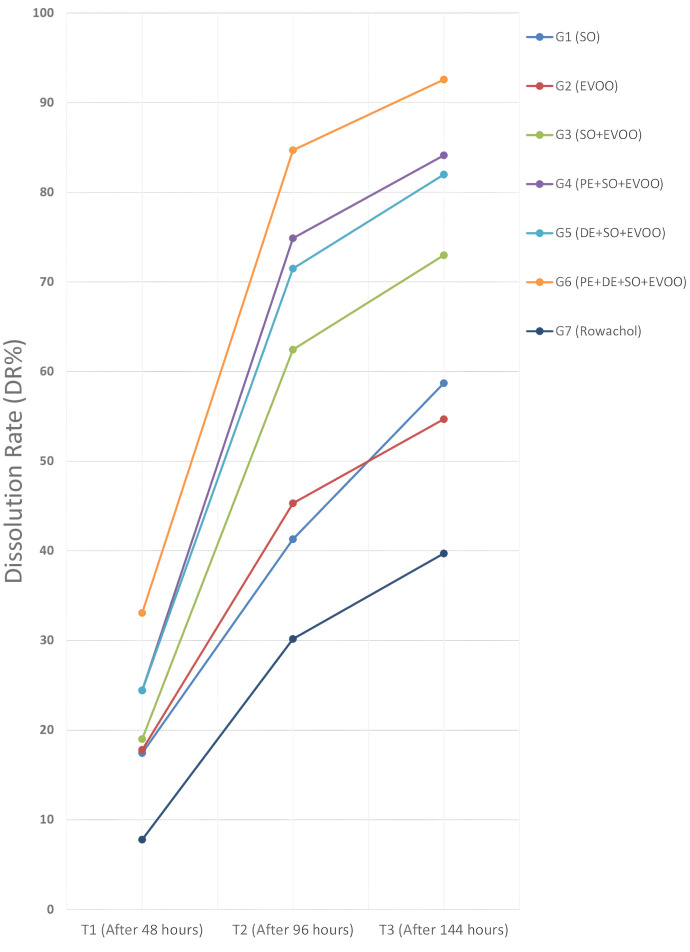
Dissolution rate (DR, %) across groups over time (T0–T3). Line plots of DR (%) at T0 (0 h), T1 (48 h), T2 (96 h), and T3 (144 h) for all groups: G1 (SO), G2 (EVOO), G3 (SO+EVOO), G4 (PE+SO+EVOO), G5 (DE+SO+EVOO), G6 (PE + DE+SO+EVOO), and G7 (Rowachol®). Group G6 showed the highest DR by T3 (92.57%), followed by G4 (84.12%) and G5 (81.98%), whereas Rowachol® (G7) reached 39.71%. Values shown represent group means; measurement procedures are detailed in Methods **Source**: Created by the authors. G: group; **GS**: gallstones; **EVOO**: Extra Virgin Olive oil; **PE**: Psyllium extract; **SO**: Sesame oil. **Source**: Created by the authors.

**Fig 5:**
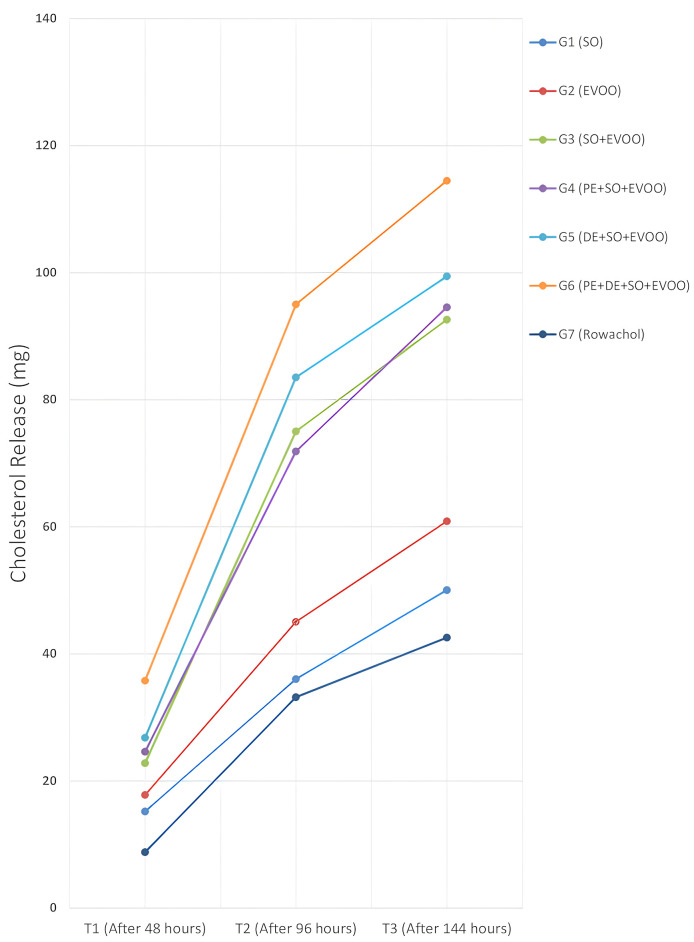
Cumulative cholesterol released (mg) over time (T1–T3). Line plots of cumulative cholesterol release (mg) at T1 (48 h), T2 (96 h), and T3 (144 h) across all groups: G1 (SO), G2 (EVOO), G3 (SO+EVOO), G4 (PE+SO+EVOO), G5 (DE+SO+EVOO), G6 (PE + DE+SO+EVOO), and G7 (Rowachol®). By T3, G6 (PE + DE+SO+EVOO) showed the highest cumulative release (114.48 mg), followed by G5 (99.43 mg) and G4 (94.57 mg), while Rowachol® (G7) reached 42.57 mg. Values represent group means under identical incubation conditions; analytical details are provided in Methods. **Source**: Created by the authors. **G**: group; **GS**: gallstones; **EVOO**: Extra Virgin Olive oil; **PE**: Psyllium extract; **SO**: Sesame oil; **T1**: after 48 hours; **T2**: after 96 hours; **T3**: after 144 hours.

Temporal analysis (T0–T3) demonstrated progressive reductions in gallstone weight and increases in dissolution rate (DR%) and cholesterol release. All measurements exhibited high intraclass correlation coefficients (ICC: 0.93–0.97), indicating robust reproducibility (**Table 2**, **Figs 4 and 5**).

**Table 2:** Temporal Evolution of GSs Characteristics.

Group (N = 70)		Weight (mg) (Mean ± SD)				Dissolution Rate (DR%) (Mean ± SD)			Cholesterol Release (mg) (Mean ± SD)			Mean ICC
		T0	T1	T2		T3	T1	T2	T3	T1	T2	T3
**Experimental Groups**	G1 _(SO)_	121.24 ± 1.8	100.12 ± 1.6	71.18 ± 1.4	50.06 ± 1.2	17.44 ± 1.2	41.29 ± 1.7	58.71 ± 2.1	15.20 ± 1.2	36.04 ± 1.8	50.06 ± 2.1	0.96
	G2 _(EVOO)_	134.37 ± 2.3	110.45 ± 2.1	73.48 ± 1.9	60.89 ± 1.7	17.79 ± 1.4	45.31 ± 1.9	54.69 ± 2.5	17.80 ± 1.4	45.06 ± 2.1	60.89 ± 2.5	0.95
	G3 _(SO+EVOO)_	148.29 ± 2.9	120.12 ± 2.6	55.67 ± 2.2	40.06 ± 1.9	19.00 ± 1.8	62.45 ± 2.4	72.98 ± 3.3	22.80 ± 1.8	75.02 ± 3.1	92.62 ± 3.3	0.96
	G4 _(PE+SO+EVOO)_	126.31 ± 2.1	95.45 ± 1.9	31.74 ± 1.5	20.06 ± 1.3	24.42 ± 1.6	74.87 ± 2.8	84.12 ± 3.1	24.60 ± 1.6	71.87 ± 2.9	94.57 ± 3.1	0.97
	G5 _(DE+SO+EVOO)_	139.07 ± 2.5	105.12 ± 2.2	39.64 ± 1.8	25.06 ± 1.5	24.42 ± 1.7	71.49 ± 2.6	81.98 ± 3.5	26.80 ± 1.7	83.52 ± 3.4	99.43 ± 3.5	0.97
	G6 _(PE + DE+SO+EVOO)_	135.16 ± 2.2	90.45 ± 1.9	20.68 ± 1.4	10.06 ± 1.1	33.07 ± 2.1	84.69 ± 3.1	92.57 ± 4.2	35.80 ± 2.1	95.02 ± 4.0	114.48 ± 4.2	0.93
**Control Group**	G7 _(Rowachol_)	141.09 ± 2.7	130.12 ± 2.4	98.52 ± 2.1	85.06 ± 1.8	7.78 ± 0.9	30.17 ± 1.5	39.71 ± 1.9	8.80 ± 0.9	33.20 ± 1.6	42.57 ± 1.9	0.94

GSs: gallstones; G: Group; N: number of gallstones; SO: Sesame Oil; EVOO: Extra Virgin Olive Oil; PE: Psyllium Extract; DE: Dandelion Extract; T0: Baseline measurement.; T1: Measurement at 48 hours; T2: Measurement at 96 hours; T3: Measurement at 144 hours; mg: milligrams; DR: Dissolution Rate; SD: Standard Deviation; ICC: Intraclass Correlation Coefficient.

Analysis shows that Group G6 _(PE+DE+SO+EVOO)_ achieved the highest values at T3, with a dissolution rate of 92.57 ± 4.2% and cholesterol release of 114.48 ± 4.2 mg. In contrast, Group G7 _(Rowachol)_ recorded the lowest values (39.71 ± 1.9% and 42.57 ± 1.9 mg) (**Figs 4 and 5**). Moreover, ICC values for weight and cholesterol exceeded 0.9 across all groups, confirming excellent measurement reliability.

### 3.3. Comparative analysis of treatment groups

Repeated measures ANOVA showed significant effects of time (F = 85.6, p < 0.001), group (F = 42.3, p < 0.001), and their interaction (F = 12.4, p < 0.001).

The dissolution rate (DR%) at T3 was significantly higher in G6 compared to all other groups, including the control (G7: p < 0.001), G1 (p < 0.001), G2 (p < 0.001), G3 (p = 0.001), G4 (p = 0.03), and G5 (p = 0.01), as confirmed by post-hoc tests. Integrated formulations (G3–G6) outperformed the control (G7) in DR%. G1 and G2, though less effective, still showed higher DR% than G7 at T3. Full details are provided in **Table 3**.

**Table 3:** Group and Time Effects on Dissolution Rate.

Comparison N = 70		T1 (48h)		T2 (96h)		T3 (144h)	
		(DR%) Difference [CI 95%]	p-value	(DR%) Difference [CI 95%]	p-value	(DR%) Difference [CI 95%]	p-value
**G6**_**(DE + PE+SO+EVOO)**_ _**VS**_	**G7 **_**(Rowachol)**_	+25.29% [21.1, 29.4]	<0.001*	+54.52% [49.8, 59.2]	<0.001*	+52.86% [48.1, 57.6]	<0.001*
	**G1**_**(SO)**_	+15.63% [11.2, 20.0]	0.02*	+43.40% [38.5, 48.3]	<0.001*	+33.86% [29.0, 38.7]	<0.001*
	**G2**_**(EVOO)**_	+15.28% [10.8, 19.7]	0.04*	+39.38% [34.5, 44.2]	<0.001*	+37.88% [33.0, 42.7]	<0.001*
	**G3**_**(SO+EVOO)**_	+14.07% [9.6, 18.5]	0.04*	+22.24% [17.5, 27.0]	0.003*	+19.59% [14.8, 24.4]	0.001*
	**G4**_**(DE+SO+EVOO)**_	+8.65% [4.0, 13.3]	0.12 (NS)	+9.82% [5.0, 14.6]	0.09 (NS)	+8.45% [3.6, 13.3]	0.03*
	**G5**_**(PE+SO+EVOO)**_	+8.65% [4.0, 13.3]	0.12 (NS)	+13.20% [8.4, 18.0]	0.02*	+10.59% [5.7, 15.5]	0.01*
**G5**_**(PE+SO+EVOO)**_ _**VS**_	**G7 **_**(Rowachol)**_	+16.64% [12.3, 21.0]	0.002*	+41.32% [36.4, 46.2]	<0.001*	+42.27% [37.4, 47.1]	<0.001*
	**G1**_**(SO)**_	+7.00% [2.5, 11.5]	0.15 (NS)	+30.20% [25.3, 35.1]	<0.001*	+23.27% [18.5, 28.1]	<0.001*
	**G2**_**(EVOO)**_	+6.63% [2.1, 11.1]	0.18 (NS)	+26.18% [21.3, 31.0]	<0.001*	+27.29% [22.4, 32.1]	<0.001*
	**G3**_**(SO+EVOO)**_	+5.42% [0.9, 10.0]	0.25 (NS)	+9.04% [4.2, 13.9]	0.12 (NS)	+9.00% [4.1, 13.9]	0.10 (NS)
	**G4**_**(DE+SO+EVOO)**_	0.00% [-4.5, 4.5]	1.00 (NS)	+3.38% [-1.4, 8.2]	0.65 (NS)	+2.14% [-2.7, 6.9]	0.70 (NS)
**G4**_**(DE+SO+EVOO)**_ _**VS**_	**G7 **_**(Rowachol)**_	+16.64% [12.3, 21.0]	0.002*	+44.70% [39.8, 49.6]	<0.001*	+44.41% [39.5, 49.3]	<0.001*
	**G1**_**(SO)**_	+7.00% [2.5, 11.5]	0.15 (NS)	+33.58% [28.7, 38.4]	<0.001*	+25.41% [20.6, 30.2]	<0.001*
	**G2**_**(EVOO)**_	+6.63% [2.1, 11.1]	0.18 (NS)	+29.56% [24.7, 34.4]	<0.001*	+29.43% [24.6, 34.3]	<0.001*
	**G3**_**(SO+EVOO)**_	+5.42% [0.9, 10.0]	0.25 (NS)	+12.42% [7.6, 17.2]	0.06 (NS)	+11.14% [6.3, 16.0]	0.04*
**G3**_**(SO+EVOO)**_ _**VS**_	**G7 **_**(Rowachol)**_	+11.22% [6.8, 15.6]	0.09 (NS)	+32.28% [27.4, 37.1]	0.002*	+33.27% [28.4, 38.1]	<0.001*
	**G1**_**(SO)**_	+1.56% [-3.0, 6.1]	0.80 (NS)	+21.16% [16.3, 26.0]	0.001*	+14.27% [9.4, 19.1]	0.008*
	**G2**_**(EVOO)**_	+1.21% [-3.3, 5.7]	0.85 (NS)	+17.14% [12.3, 22.0]	0.004*	+18.29% [13.4, 23.2]	<0.001*
**G2**_**(EVOO)**_ _**VS**_	**G7 **_**(Rowachol)**_	+10.01% [5.6, 14.4]	0.12 (NS)	+15.14% [10.3, 20.0]	0.03*	+14.98% [10.1, 19.9]	0.04*
	**G1**_**(SO)**_	+0.35% [-4.1, 4.8]	0.98 (NS)	+4.02% [-0.8, 8.8]	0.45 (NS)	+4.00% [-0.8, 8.8]	0.45 (NS)
**G1**_**(SO)**_ _**VS**_	**G7 **_**(Rowachol)**_	+9.66% [5.2, 14.1]	0.15 (NS)	+11.12% [6.3, 15.9]	0.09 (NS)	+19.00% [14.1, 23.9]	0.02*

G: Group; N: number of gallstones; SO: Sesame Oil; EVOO: Extra Virgin Olive Oil; PE: Psyllium Extract; DE: Dandelion Extract; DR: Dissolution Rate; SD: Standard Deviation; T1: Measurement at 48 hours; T2: Measurement at 96 hours; T3: Measurement at 144 hours; h: hours; CI: Confidence Interval; *: Tukey’s HSD test; NS: Not significant.

Cholesterol release increased significantly over time and differed among groups (p < 0.001). Integrated formulations (G3–G6) released more cholesterol than the control (G7) at all time points. At T3, G6 released +71.91 mg more than G7 (p < 0.001) and outperformed G1 (+64.42 mg, p < 0.001), G2 (+53.61 mg, p < 0.001), G3 (+21.86 mg, p = 0.002), G4 (+19.91 mg, p = 0.03), and G5 (+15.05 mg, p = 0.02). Additionally, G4 released less cholesterol than G5 at T2 (p = 0.01) and T3 (p = 0.04), confirming the superior performance of the integrated formulations (**Table 4**).

**Table 4:** Group and Time Effects on Cholesterol Release.

Comparison N = 70		T1 (48h)		T2 (96h)		T3 (144h)	
		Difference (mg) [CI 95%]	p-value	Difference (mg) [CI 95%]	p-value	Difference (mg) [CI 95%]	p-value
**G6**_**(DE + PE+SO+EVOO)**_ _**VS**_	**G7 **_**(Rowachol)**_	+27.00 [24.1, 29.9]	<0.001*	+61.82 [56.3, 67.3]	<0.001*	+71.91 [65.2, 78.6]	<0.001*
	**G1**_**(SO)**_	+20.60 [17.2, 24.0]	<0.001*	+58.98 [53.5, 64.4]	<0.001*	+64.42 [58.0, 70.8]	<0.001*
	**G2**_**(EVOO)**_	+18.00 [14.6, 21.4]	<0.001*	+49.96 [44.6, 55.3]	<0.001*	+53.61 [47.3, 59.9]	<0.001*
	**G3**_**(SO+EVOO)**_	+13.00 [9.5, 16.5]	0.001*	+20.00 [14.8, 25.2]	0.003*	+21.86 [15.7, 28.0]	0.002*
	**G4**_**(DE+SO+EVOO)**_	+11.20 [7.6, 14.8]	0.01*	+23.15 [17.9, 28.4]	0.02*	+19.91 [13.6, 26.2]	0.03*
	**G5**_**(PE+SO+EVOO)**_	+9.00 [5.3, 12.7]	0.04*	+11.50 [6.3, 16.7]	0.05 (NS)	+15.05 [8.7, 21.4]	0.02*
**G5**_**(PE+SO+EVOO)**_ _**VS**_	**G7 **_**(Rowachol)**_	+18.00 [15.1, 20.9]	<0.001*	+50.32 [45.5, 55.1]	<0.001*	+59.95 [54.2, 65.7]	<0.001*
	**G1**_**(SO)**_	+11.60 [8.3, 14.9]	<0.001*	+47.48 [42.4, 52.5]	<0.001*	+52.46 [46.5, 58.4]	<0.001*
	**G2**_**(EVOO)**_	+9.00 [5.6, 12.4]	0.002*	+39.26 [34.2, 44.3]	<0.001*	+41.05 [35.1, 47.0]	<0.001*
	**G3**_**(SO+EVOO)**_	+4.00 [0.5, 7.5]	0.04*	+14.82 [9.7, 19.9]	0.001*	+9.00 [2.9, 15.1]	0.03*
	**G4**_**(DE+SO+EVOO)**_	−2.20 [-5.6, 1.2]	0.25 (NS)	−11.65 [-16.8, -6.5]	0.01*	−7.95 [-14.1, -1.8]	0.04*
**G4**_**(DE+SO+EVOO)**_ _**VS**_	**G7 **_**(Rowachol)**_	+15.80 [12.9, 18.7]	<0.001*	+38.67 [33.9, 43.4]	<0.001*	+52.00 [46.3, 57.7]	<0.001*
	**G1**_**(SO)**_	+9.40 [6.1, 12.7]	0.004*	+35.83 [30.8, 40.8]	<0.001*	+44.51 [38.6, 50.4]	<0.001*
	**G2**_**(EVOO)**_	+6.80 [3.4, 10.2]	0.03*	+26.81 [21.7, 31.9]	<0.001*	+33.10 [27.2, 39.0]	<0.001*
	**G3**_**(SO+EVOO)**_	+1.80 [-1.7, 5.3]	0.45 (NS)	+3.15 [-2.0, 8.3]	0.32 (NS)	+1.05 [-5.0, 7.1]	0.80 (NS)
**G3**_**(SO+EVOO)**_ _**VS**_	**G7 **_**(Rowachol)**_	+14.00 [11.1, 16.9]	<0.001*	+41.82 [37.0, 46.6]	<0.001*	+50.95 [45.2, 56.7]	<0.001*
	**G1**_**(SO)**_	+7.60 [4.3, 10.9]	0.001*	+38.98 [33.9, 44.0]	<0.001*	+42.95 [37.0, 48.9]	<0.001*
	**G2**_**(EVOO)**_	+5.00 [1.6, 8.4]	0.03*	+30.82 [25.7, 35.9]	<0.001*	+33.05 [27.1, 39.0]	<0.001*
**G2**_**(EVOO)**_ _**VS**_	**G7 **_**(Rowachol)**_	+9.00 [6.1, 11.9]	<0.001*	+11.00 [6.3, 15.7]	0.002*	+17.90 [12.1, 23.7]	<0.001*
	**G1**_**(SO)**_	+2.60 [-0.7, 5.9]	0.15 (NS)	+9.02 [4.0, 14.0]	0.04*	+10.83 [4.9, 16.8]	0.02*
**G1**_**(SO)**_ _**VS**_	**G7 **_**(Rowachol)**_	+6.40 [3.5, 9.3]	0.004*	+1.98 [-3.0, 6.9]	0.55 (NS)	+7.07 [1.2, 12.9]	0.04*

G: Group; N: number of gallstones; SO: Sesame Oil; EVOO: Extra Virgin Olive Oil; PE: Psyllium Extract; DE: Dandelion Extract; mg: milligrams; SD: Standard Deviation; T1: Measurement at 48 hours; T2: Measurement at 96 hours; T3: Measurement at 144 hours; h: hours; CI: Confidence Interval; *: Tukey’s HSD test; NS: Not significant.

### 3.4. Synergistic effects of the natural formulations

The synergistic effects of mixed groups were evident at T3 (Table 5). The combination of SO and EVOO (G3) significantly enhanced dissolution rate (+16.28%, p < 0.01) and cholesterol release (+37.15 mg, p < 0.01) compared to individual oils (G1 + G2). Similarly, the combined herbal extract and oil group (G6) showed greater dissolution (+9.52%, p = 0.02) and cholesterol release (+17.48 mg, p = 0.03) than the summed individual herbal groups (G4 + G5). All differences were statistically significant (p < 0.05), supporting synergistic interactions between components.

**Table 5:** Synergistic Effects of Mixed vs. Individual Groups on Dissolution and Cholesterol Release at T3.

Comparison	Mixed Group (N = 10)	Individual Group (N = 20)	Dissolution Rate (DR%)		Cholesterol Release (mg)	
			Difference [CI 95%]	p-value *	Difference [CI 95%]	p-value*
**G3**_**(SO+EVOO)**_ **vs.** **G1**_**(SO)**_ **+G2**_**(EVOO)**_	G3	G1 + G2	**+16.28** [13.55, 19.01]	**<0.01**	**+37.15** [34.42, 39.87]	**<0.01**
**G6**_**(DE + PE+SO+EVOO)**_ **vs.** **G4**_**(DE+SO+EVOO)**_ **+G5**_**(PE+SO+EVOO)**_	G6	G4 + G5	**+9.52** [5.96, 13.08]	**0.02**	**+17.48** [13.92, 21.04]	**0.03**

T3: Measurement at 144 hours; G: Group; N: number of gallstones; SO: Sesame Oil; EVOO: Extra Virgin Olive Oil; PE: Psyllium Extract; DE: Dandelion Extract; mg: milligrams; DR: Dissolution Rate; SD: Standard Deviation; CI: Confidence Interval; *: Two-sample t-test with Welch’s correction for unequal variances.

### 3.5. Correlation between chemical composition and dissolution efficacy

Correlation analysis demonstrated significant positive associations between specific chemical compounds and both gallstone dissolution rate (DR%) and cholesterol release (Table 6). Oleic acid exhibited the strongest correlations (DR%: r = +0.89, p < 0.001; cholesterol release: r = +0.92, p < 0.001), followed by hydroxytyrosol (r = +0.82) and oleuropein (r = +0.78–0.80). Among plant extracts, taraxacin showed the highest efficacy (DR%: r = +0.91; cholesterol release: r = +0.94), with arabinoxylan (r = +0.85) and flavonoids (r = +0.79) also displaying robust effects. In contrast, palmitic acid, sesamin, and bitter compounds revealed no statistically significant relationships (p > 0.05).

**Table 6:** Correlation between chemical composition and gallstone dissolution outcomes.

A. Oils				
Compound	Correlation* (DR%)	p-value	Correlation* (Cholesterol release)	p-value
Oleic Acid	+0.89	<0.001	+0.92	<0.001
Linoleic Acid	+0.76	0.004	+0.81	0.001
Palmitic Acid	+0.12	0.75	+0.09	0.82
Hydroxytyrosol	+0.82	0.002	+0.85	0.001
Oleuropein	+0.78	0.005	+0.80	0.003
Sesamin	−0.15	0.70	−0.10	0.80
Vitamin E	+0.68	0.02	+0.72	0.01
**B. Extracts**				
Arabinoxylan	+0.85	0.001	+0.88	<0.001
Taraxacin	+0.91	<0.001	+0.94	<0.001
Flavonoids	+0.79	0.004	+0.82	0.002
Polyphenols	+0.73	0.01	+0.76	0.008
Bitter Compounds	+0.45	0.18	+0.50	0.12

DR: Dissolution Rate; *: Pearson’s correlation.

### 3.6. Predictive factors of gallstone dissolution efficacy

Multiple regression analysis identified oleic acid (β = 0.85, p = 0.002), taraxacin (β = 0.92, p < 0.001), and arabinoxylan (β = 0.78, p = 0.005) as the strongest predictors of gallstone dissolution efficacy (Table 7). Linoleic acid showed a smaller, moderate effect (β = 0.18, p = 0.03). These variables collectively explained 94% of the variance in outcomes (adjusted R² = 0.94), highlighting the critical role of monounsaturated fatty acids (e.g., oleic acid in EVOO) and bioactive plant compounds (e.g., taraxacin in dandelion) in enhancing dissolution.

**Table 7:** Multiple Regression Analysis of Factors Influencing Gallstone Dissolution Efficacy.

Independent Variable	Regression Coefficient (β)	p-value *	Statistical Significance
Oleic Acid	0.85	0.002	strong significance
Taraxacin	0.92	<0.001	very strong significance
Arabinoxylan	0.78	0.005	strong significance
Linoleic Acid	0.18	0.03	moderate significance
**Adjusted R²**	**0.94**		

*: Multiple linear regression analysis.

## 4. Discussion

Medicinal plants have long played a central role in healthcare, including in the Middle East, where herbal remedies are widely used for hepatobiliary disorders [27]. In this randomized in-vitro study, the four-component combination of sesame oil (SO), extra virgin olive oil (EVOO), psyllium extract (PE), and dandelion extract (DE) (G6) achieved a mean dissolution rate (DR) of 92.57% ± 4.2 and a mean cumulative cholesterol release of 114.48 ± 4.2 mg at 144 h (T3), significantly outperforming all comparators, including Rowachol® (G7) and the other experimental groups (G1–G5).The effect was time-dependent, with progressive separation from T1 to T3. Exploratory chemistry–outcome analyses indicated positive associations between key bioactives (oleic acid, taraxacin, arabinoxylan, linoleic acid) and dissolution metrics, suggesting a greater-than-additive contribution under the tested conditions. Given the short-term, controlled in-vitro nature of these experiments, the findings should be interpreted as mechanistic signals rather than clinical guidance.

### 4.1. Natural agents for gallstone dissolution: Rationale & mechanisms

This study’s use of natural oils and herbal extracts aligns with prior research highlighting their therapeutic potential [24]. Olive oil contains oleic acid, phenolic compounds (e.g., hydroxytyrosol, oleuropein), and vitamin E [28], while sesame oil provides sesamin, sesamolin, phytosterols, and vitamin E [29]; these compounds collectively enhance gallbladder contraction, reduce inflammation, lower cholesterol, and exert antioxidant effects [30]. Dandelion extracts, rich in flavonoids, phenolic acids, and sesquiterpene lactones, demonstrate antioxidant and anti-inflammatory properties that may reduce inflammation and promote gallstone breakdown [31,32]. Psyllium, a source of soluble fiber via mucilage polysaccharides like arabinoxylans and xyloglucans, regulates cholesterol, improves bile acid metabolism, and aids in gallstone prevention/dissolution [33,34].

### 4.2. Rowachol® as a pharmacological benchmark

Rowachol®, a terpene-based cholelitholytic agent, is clinically used for gallstone dissolution. It combines peppermint, fir, eucalyptus, rice, anise, and extra virgin olive oils to enhance bile secretion, break down gallstones, reduce biliary inflammation, and improve digestion [35]. Its mechanism involves inhibiting hepatic HMG-CoA reductase, altering biliary cholesterol saturation, and lowering bile lithogenicity. Menthol, a key component, is secreted as menthol glucuronide in bile, enhancing calcium carbonate/phosphate solubility to reduce gallstone calcification. Additionally, Rowachol’s components exhibit antioxidant, antiseptic, antispasmodic, and analgesic effects. Clinical studies support its efficacy in improving gallstone outcomes with long-term use [35].

### 4.3. Impact of time on gallstone dissolution rate and cholesterol release

The results demonstrate that time critically enhances gallstone dissolution and cholesterol release. Initially hindered by the gallstone’s crystalline structure, solvent penetration improves over time, enabling chemical interactions that reduce gallstone weight by breaking cholesterol bonds. This progressive dissolution peaks in G6_(PE+DE+SO+EVOO)_, showing a 2.8-fold increase in dissolution rate (from 33.07% at T1 to 92.57 ± 4.2% at T3) and a 3.2-fold surge in cholesterol release (from 35.80 mg at T1 to 114.48 ± 4.2 mg at T3). Notably, G6’s time-dependent efficacy far surpassed the control group (G7_Rowachol_), which achieved only 39.71% dissolution and 42.57 mg cholesterol release at T3. These findings align with **B.K. et al.** [25], who noted time-dependent efficacy, with cholesterol-rich stones dissolving faster in lipid solvents compared to pigment stones requiring prolonged exposure due to their non-polar composition. **Tiwari and Sah** [23] further linked prolonged exposure and higher extract concentrations to increased cholesterol release, driven by the law of mass action. Mechanistically, extended time allows active molecules to penetrate the gallstone surface, inducing gradual chemical and physical changes. In contrast, pigment stones resist these effects, underscoring the need for type-specific therapies based on gallstone composition.

### 4.4. Molecular synergy: Mechanism of compound interaction in enhancing gallstone dissolution

A synergistic effect was anticipated because each natural agent addresses a different rate-limiting step in cholesterol-stone dissolution. First, the unsaturated fatty acids in extra-virgin olive oil and sesame oil (notably oleic and linoleic acids) enlarge the cholesterol-carrying capacity of bile-salt micelles and loosen the crystal lattice, a phenomenon demonstrated by classic and recent micellar-solubility studies [29].

Second, psyllium’s arabinoxylan fiber binds bile acids in the intestinal lumen, accelerating their hepatic re-synthesis and thereby sustaining a low, unsaturated biliary cholesterol index; contemporary work also shows that psyllium-driven shifts in bile-acid pools activate FXR signaling and enhance cholesterol export [36,37].

Third, dandelion root and leaf extracts contain bitter sesquiterpene lactones (e.g., taraxacin) that stimulate choleresis, increasing bile flow and mechanically renewing the solvent around the stone surface [38,39].

Finally, sesame lignans (sesamin, sesamol) supply potent antioxidants that limit oxidative cross-linking within partially dissolved stones, preventing re-hardening and permitting deeper penetration of the solvent front [29,40].

When combined, these complementary actions create a self-reinforcing cycle: unsaturated lipids destabilize the crystal core; increased bile turnover and flow continually clear dissolved cholesterol; and antioxidant protection keeps the matrix receptive to further attack. The concept aligns with emerging clinical evidence that multi-component regimens outperform single-agent therapy, for example, the superior dissolution seen with polyunsaturated fatty acids plus ursodeoxycholic acid compared with ursodeoxycholic acid alone [41].

Thus, the present formulation was assembled to reproduce, in one mixture, a cascade of mechanisms that individually aid dissolution but together deliver the marked (>90%) weight loss observed in vitro.

**Chekroune and Benamara** [21] reported 100% gallstone dissolution within 7 days using an olive oil/lemon juice mixture, attributing this to emulsion-enhanced lipid-aqueous interactions and improved permeability. While their study lacked molecular analysis (e.g., limonene’s role), the current study identifies how specific compounds disrupt gallstone architecture, advancing molecular-level strategies for cholesterol gallstone therapy.

### 4.5. The dual nature of sesame oil in gallstone management: Prevention over dissolution

The results demonstrate significant variations in gallstone dissolution efficacy among tested materials, with G1_Sesame oil_ exhibiting the lowest rates across all time intervals. This outcome stems from sesame oil’s unique physicochemical profile: while its phenolic antioxidants (e.g., sesamin, sesamole) inhibit hepatic cholesterol synthesis [42], they primarily prevent new stone formation rather than dissolving existing ones. Physically, sesame oil’s high viscosity impedes penetration into gallstone micro-pores [43], and its hydrophobic nature limits interaction with semi-polar cholesterol surfaces [44]. Mechanistically, unlike EVOO—whose free fatty acids directly react with cholesterol—sesame oil’s fatty acids remain bound in triglycerides, reducing dissolution capacity [2]. Minimal improvement over time (T0–T3) suggests prolonged exposure or optimal conditions may enhance efficacy, consistent with studies showing sesamin requires extended periods to degrade cholesterol bonds [45]. Sesame oil’s limited dissolution efficacy reflects its distinct preventive role (via cholesterol reduction) rather than therapeutic weakness.

### 4.6. Natural agents in gallstone management: A comprehensive analysis of in vitro evidence

Emerging in vitro studies demonstrate the gallstone-dissolving potential of natural compounds through distinct mechanisms. Chekroune and Benamara [21] achieved complete dissolution using an olive oil-lemon juice emulsion, combining oleic acid’s cholesterol-solubilizing action with lemon’s acidity to enhance bile salt activity. Sulaiman [22] observed barley water extract’s structural reduction via beta-glucan’s cholesterol-binding and phenolics’ crystalline disruption. Tiwari and Sah [23] reported apricot extracts releasing 377.3 mg/dL cholesterol, mediated by amygdalin and chlorogenic acid’s competitive displacement of cholesterol from bile salt micelles. Arrout et al. [24] highlighted Moroccan citrus oil’s superiority, with limonene penetrating cholesterol matrices and linalool reducing bile viscosity. B.K et al. [25] identified *Berberis asiatica*’s calcium-targeting alkaloids for mixed stones versus *Taraxacum*’s bilirubin-disrupting triterpenes for pigment stones, while Nodehi et al. [26] demonstrated chicory and wormwood degrade lipids via sesquiterpene lactones and inulin’s synergistic hydrolysis. Though these studies validate individual mechanisms, they neglect combination effects. This study is the first in vitro comparison of oils (sesame, extra virgin olive) and herbs (psyllium, dandelion) against Rowachol® for cholesterol gallstone dissolution.

### 4.7. Limitations

While this study demonstrates the superior efficacy of the quadruple natural combination (sesame oil, EVOO, psyllium, dandelion) over pharmaceutical controls, certain constraints warrant mention. The in vitro model, though methodologically rigorous, lacks physiological elements of human biliary systems (e.g., bile flow dynamics, pH variations, immune responses), potentially limiting clinical extrapolation. The exclusive focus on cholesterol gallstones restricts applicability to pigment or mixed-composition stones with differing dissolution mechanisms. The 144-hour experimental period, while sufficient for initial dissolution assessment, may not reflect in vivo timelines for larger stones. Fixed-dose administration (100 mg/mL) overlooks dose-response variability and interpatient heterogeneity in metabolism or comorbidities. Furthermore, cytotoxicity and cellular safety profiles remain unexamined, as the study prioritized dissolution efficacy.

### 4.8. Clinical relevance and translation

The present findings were obtained under simplified in-vitro conditions that do not replicate the complexity of the biliary environment, gallbladder motility, or inter-patient variability. Moreover, safety, tolerability, and optimal dosing were not assessed. Consequently, these results should not be extrapolated to clinical recommendations. Instead, they support prioritizing in-vivo validation to characterize pharmacodynamics, toxicity, and dosing, followed by rigorously designed clinical trials to determine real-world effectiveness.

### 4.9. Future directions

Subsequent research should prioritize in-vivo models to evaluate physiological determinants (e.g., bile flow and composition, gallbladder motility, immune interactions) and to perform dose-ranging studies that define therapeutic windows relevant to clinical translation. Comparative analyses across gallstone subtypes (cholesterol vs. pigment) are warranted to refine target populations. Comprehensive safety profiling—including hepatocyte and biliary epithelial viability, liver function markers, and histopathology—should be conducted to establish toxicity thresholds. Studies on potential herb–drug interactions (e.g., with anticoagulants or statins) and health-economic evaluations against conventional therapies will further clarify translational value.

## 5. Conclusions

The study results indicate that combining sesame oil, extra virgin olive oil, psyllium extract, and dandelion extract offers a promising treatment for dissolving cholesterol gallstones. Below are the key findings:

1 **Strongest Efficacy with the Four-Component Combination**

- The integrated combination of the four substances achieved the **highest dissolution rate and cholesterol release**, outperforming the pharmaceutical control (Rowachol®) by **2.3- 2.7-fold,** respectively.
- This success stems from a **synergistic molecular interaction** that enhances the substances’ ability to penetrate and dismantle the gallstone structure.

2 **Time-Dependent Efficacy**

- The study revealed a **significant increase** in gallstone dissolution and cholesterol release over time.
- Results confirm that **time is critical**: prolonged exposure to the compounds amplifies therapeutic outcomes, underscoring the importance of cumulative interactions in disrupting cholesterol crystals.

3 **Molecular Synergy of Active Compounds**

- The study highlights four compounds as key synergistic agents:
  - **Oleic Acid** (from olive oil)
  - **Taraxacin** (from dandelion)
  - **Arabinoxylan** (from psyllium)
  - **Linoleic Acid** (from sesame oil)
- The integrated interaction of these compounds demonstrates their **harmonized action** in enhancing gallstone dissolution.

4 **Preventive Role of Sesame Oil**

- While sesame oil showed lower dissolution efficacy compared to olive oil when used alone, it exhibits **important preventive properties**.
- Sesame oil contains antioxidants and cholesterol-reducing compounds, making it a **strategic component** in therapeutic and preventive protocols.

5 **New Horizons in Gallstone Management**

- The study proves that **nature can provide multi-path solutions** that surpass single-target pharmaceuticals.
- It highlights the **vast potential** of natural combinations as innovative, safe alternatives to traditional drug therapies like Rowachol®.
- The clear evidence of time-dependent cholesterol release and dissolution rate improvements opens avenues for applying this strategy in **future clinical trials**.

In conclusion, combining EVOO, SO, psyllium, and dandelion extracts improved cholesterol gallstone dissolution under controlled in-vitro conditions. These findings provide mechanistic support for further investigation. Future in-vivo studies are necessary to define safety, dosing, and biological plausibility in living systems, followed by clinical trials to evaluate real-world effectiveness before any consideration of patient-care application.

## Supporting information

S1 Table Mechanisms of Sesame Oil, Olive Oil, Psyllium, and Dandelion on Gallstones. (DOCX)

S2 Table Comprehensive Protocol for Sourcing, Extraction, and Preservation of Nutrient Oils and Plant Extracts. (DOCX)

S3 Table Chemical Composition and Gallstone-Dissolving Mechanisms of Plant Oils and Extracts. (DOCX)

S4 Table Extraction Protocol of 100 mg Terpenes from Rowachol® Capsules. (DOCX)

S5 Table Specifications of BioIVT Bile Juice Used in the Study. (DOCX)

S6 Table Mixing and Dissolution Procedures for Groups. (DOCX)

## List of Abbreviations:

AIU: Arab International University
DE: Dandelion Extract
DR: Dissolution Rate
G: Group
GS: Gallstones
EVOO: Extra Virgin Olive Oil
PE: Psyllium Extract
DE: Dandelion Extract
SO: Sesame Oil
MTBE: methyl tertbutyl ether
DR%: Dissolution Rate
ICC: Intraclass Correlation Coefficient
ANOVA: Analysis of Variance
FTIR: Fourier-Transform Infrared Spectroscopy
GC-MS: Gas Chromatography-Mass Spectrometry
HPLC: High-Performance Liquid Chromatography
BMI: Body Mass Index
ERCP: Endoscopic Retrograde Cholangiopancreatography
ESWL: Extracorporeal Shock Wave Lithotripsy
FDA: Food and Drug Administration
R²: Coefficient of Determination
T0: before the experiment
T1: after 48 hours
T2: after 96 hours
T3: After 144 hours.
