# Supplementary material for: Synergistic effects of sesame oil, extra virgin olive oil, psyllium extract, and dandelion extract on cholesterol gallstone dissolution: An in vitro comparative study against Rowachol®
Source: PLoS One. 2025 Oct 14;20(10):e0334496. doi: 10.1371/journal.pone.0334496 (PMC12520339; doi:10.1371/journal.pone.0334496)
Supplement: S1 Table — (DOCX) [file pone.0334496.s001.docx]

| **Supplementary Table 1:** Mechanisms of Sesame Oil, Olive Oil, Psyllium, and Dandelion on Gallstones | | | | |
| --- | --- | --- | --- | --- |
| **Substance** | **Benefit** | **Active Components** | **Effect on Gallbladder** | **Detailed Mechanism** |
| **Sesame Oil** | **Anti-lipogenic** | Sesamin, Sesamolin, Linoleic Acid (PUFA) | Reduces cholesterol production in the liver, lowering its concentration in bile. | - **Inhibition of HMG-CoA reductase**: The key enzyme responsible for cholesterol synthesis in the liver, reducing endogenous cholesterol production. - **Activation of PPAR-α receptors**: Enhances fatty acid oxidation in the liver, reducing triglyceride and cholesterol accumulation. - **Increased lipolysis**: Stimulates breakdown of stored fats in adipose tissue, lowering blood lipid levels. |
|  | **Hypo-cholesterolemic** | Linoleic Acid (PUFA), Sesamin, Sesamolin | Reduces cholesterol concentration in bile, lowering saturation and stone formation. | - **Increased cholesterol excretion via bile**: Stimulates cholesterol secretion from the liver to the intestines. - **Conversion of cholesterol to bile acids**: Activates liver enzymes (e.g., CYP7A1) to convert cholesterol into bile acids, reducing free cholesterol in bile. - **Reduced intestinal cholesterol absorption**: Inhibits dietary cholesterol absorption by binding to bile acids. |
|  | **Antioxidant** | Tocopherol (Vitamin E), Sesamin, Sesamolin | Protects liver and gallbladder cells from oxidative damage. | - **Neutralizes free radicals**: Scavenges reactive oxygen species (ROS) like superoxide anions (O₂⁻) and hydrogen peroxide (H₂O₂), reducing oxidative stress. - **Prevents LDL oxidation**: Reduces oxidation of LDL cholesterol, preventing its accumulation in bile. - **Activates antioxidant enzymes**: Enhances activity of glutathione peroxidase (GPx) and superoxide dismutase (SOD). |
|  | **Anti-inflammatory** | Sesamin, Sesamolin, Linoleic Acid (PUFA) | Reduces gallbladder inflammation, improving bile flow. | - **Inhibition of pro-inflammatory cytokines**: Reduces secretion of IL-6 and TNF-α. - **Inhibition of NF-κB pathway**: Suppresses inflammatory signaling. - **Activation of anti-inflammatory pathways**: Stimulates PPAR-γ to reduce inflammation and improve gallbladder function. |
|  | **Improves Bile Quality** | Linoleic Acid (PUFA), Sesamin, Sesamolin | Reduces bile stagnation, lowering stone risk. | - **Increased bile secretion**: Stimulates bile flow from the liver. - **Reduced cholesterol in bile**: Enhances cholesterol conversion to bile acids. - **Improved gallbladder motility**: Promotes regular bile emptying via gallbladder contractions. |
|  | **High Oil Stability** | Tocopherol, Sesamin, Sesamolin | Protects the liver and gallbladder by preventing harmful compound formation. | - **Antioxidant-rich composition**: Natural lignans and tocopherol prevent oil oxidation. - **Reduces oxidized lipids**: Minimizes formation of oxidized lipids that damage liver/gallbladder cells. - **Protects against oxidative damage**: Shields cells from free radical-induced damage. |
| **Olive Oil** | **Reduces Oxidative Stress** | Polyphenols (Hydroxytyrosol, Oleuropein), Vitamin E | Inhibits free radical damage to gallbladder cells. | - **Hydroxytyrosol**: Neutralizes ROS and inhibits lipid peroxidation. - **Oleuropein**: Activates Nrf2/ARE pathway, boosting antioxidant enzymes (e.g., glutathione peroxidase). - **Vitamin E**: Protects cell membranes by scavenging free radicals. |
|  | **Regulates Cholesterol Levels** | Monounsaturated Fatty Acids (Oleic Acid) | Reduces bile cholesterol saturation, limiting cholesterol stone formation. | - **Oleic Acid**: Downregulates cholesterol synthesis genes (e.g., HMG-CoA reductase). - **Activates LXR receptors**: Increases cholesterol excretion into bile. - **Enhances CYP7A1 activity**: Converts cholesterol to bile acids. |
|  | **Stimulates Bile Secretion** | Squalene, Chlorophyll | Prevents bile stagnation and cholesterol accumulation. | - **Squalene**: Activates FXR receptors to increase bile acid production. - **Chlorophyll**: Stimulates CCK hormone release, promoting gallbladder contractions. |
|  | **Inhibits Cholesterol Formation** | Polyphenols, Squalene | Reduces cholesterol deposition in the gallbladder. | - **Polyphenols**: Inhibit hepatic cholesterol synthesis enzymes. - **Squalene**: Blocks intestinal cholesterol absorption via NPC1L1 inhibition. |
|  | **Improves Gallbladder Health** | Vitamin E, Chlorophyll | Maintains gallbladder tissue integrity and function. | - **Vitamin E**: Protects cell membranes from oxidative damage. - **Chlorophyll**: Activates DNA repair pathways to regenerate damaged gallbladder cells. |
|  | **Reduces Inflammation** | Polyphenols (Oleuropein) | Prevents chronic inflammation that impairs gallbladder function. | - **Oleuropein**: Inhibits NF-κB pathway, reducing TNF-α and IL-6 secretion. - **Activates PPAR-γ**: Exerts anti-inflammatory effects. |
|  | **Enhances Fat Digestion** | Monounsaturated Fatty Acids | Improves fat digestion, reducing gallbladder pressure. | - **Oleic Acid**: Stimulates CCK release, enhancing gallbladder contractions and bile secretion. - **Promotes micelle formation**: Improves fat absorption, reducing gallbladder strain. |
| **Psyllium** | **Lowers Blood Cholesterol** | Soluble Fiber (Arabinoxylan) | Reduces risk of cholesterol gallstones. | - **Binds bile acids**: Prevents bile acid reabsorption, forcing the liver to use cholesterol to synthesize new bile acids. - **Activates 7α-hydroxylase**: Enhances cholesterol-to-bile acid conversion, reducing bile cholesterol concentration. |
|  | **Improves Intestinal Motility** | Gel-Forming Fibers | Reduces bile stagnation by enhancing gallbladder emptying. | - **Increases stool bulk**: Stimulates peristalsis and CCK secretion. - **Regulates CCK release**: Promotes regular bile flow, preventing cholesterol crystallization. |
|  | **Reduces Inflammation** | Antioxidants, Anti-inflammatory Compounds | Limits chronic inflammation in bile ducts. | - **Scavenges free radicals**: Reduces oxidative stress in gallbladder tissues. - **Produces SCFAs**: Fermentable fibers generate short-chain fatty acids (SCFAs) that suppress pro-inflammatory cytokines (e.g., TNF-α, IL-6). |
|  | **Enhances Insulin Sensitivity** | Soluble Fiber | Reduces insulin resistance, a risk factor for gallstones. | - **Slows glucose absorption**: Lowers postprandial glucose spikes, improving lipid metabolism. - **Modifies gut microbiome**: Promotes beneficial bacteria (e.g., Bifidobacteria) that metabolize cholesterol. |
| **Dandelion** | **Stimulates Bile Secretion** | Triterpenoids (Taraxasterol, Taraxerol) | Increases bile flow, reducing stagnation. | - **Activates FXR receptors**: Enhances bile acid synthesis and secretion. - **Promotes CCK release**: Triggers gallbladder contractions. |
|  | **Reduces Bile Viscosity** | Sesquiterpenoids, Flavonoids (Luteolin) | Inhibits cholesterol crystallization. | - **Modifies bile composition**: Prevents cholesterol aggregation into stones. - **Enhances bile fluidity**: Reduces viscosity through chemical modulation. |
|  | **Inhibits Stone Formation** | Phenolics (Chlorogenic Acid, Caffeic Acid) | Reduces cholesterol accumulation in bile. | - **Activates CYP7A1**: Converts cholesterol to bile acids, lowering free cholesterol levels. - **Antioxidant effects**: Prevents oxidative damage to bile components. |
|  | **Antioxidant** | Flavonoids, Vitamins (C, E) | Protects gallbladder cells from oxidative damage. | - **Neutralizes ROS**: Reduces inflammation and cellular damage linked to gallstones. - **Enhances antioxidant defenses**: Boosts endogenous enzyme activity. |
|  | **Anti-inflammatory** | Sesquiterpenoids, Coumarins | Reduces gallbladder and bile duct inflammation. | - **Inhibits NF-κB pathway**: Lowers TNF-α and IL-6 production. - **Suppresses inflammatory swelling**: Prevents bile duct obstruction. |
|  | **Regulates Lipid Levels** | Inulin, Dietary Fiber | Lowers total cholesterol and triglycerides. | - **Binds intestinal cholesterol**: Reduces absorption and bile cholesterol content. - **Promotes beneficial bacteria**: Enhances bile acid metabolism via gut microbiota modulation. |
|  | **Improves Gallbladder Motility** | Bitter Compounds (Lactucopicrin) | Stimulates regular gallbladder emptying. | - **Activates CCK receptors**: Triggers gallbladder contractions to prevent bile stagnation. |
|  | **Inhibits Digestive Enzymes** | Triterpenoids, Flavonoids | Reduces fat digestion and cholesterol availability. | - **Inhibits pancreatic lipase**: Limits breakdown of dietary fats into free fatty acids, reducing cholesterol uptake. |
|  | **Enhances Liver Function** | Polyphenols, Triterpenoids | Boosts liver detoxification processes. | - **Activates glutathione S-transferase**: Improves cholesterol and bile acid metabolism, supporting gallbladder health. |
