# Supplementary material for: Synergistic effects of sesame oil, extra virgin olive oil, psyllium extract, and dandelion extract on cholesterol gallstone dissolution: An in vitro comparative study against Rowachol®
Source: PLoS One. 2025 Oct 14;20(10):e0334496. doi: 10.1371/journal.pone.0334496 (PMC12520339; doi:10.1371/journal.pone.0334496)
Supplement: S2 Table — (DOCX) [file pone.0334496.s002.docx]

| **Supplementary Table 2:** Comprehensive Protocol for Sourcing, Extraction, and Preservation of Nutrient Oils and Plant Extracts | |
| --- | --- |
| **Process Step** | **Details** |
| **A. Nutrient Oils** | |
| **Sourcing** | - **EVOO**: from a local Hama cultivar (Syria), supplied by **Mediterranean Harvest***®,* Hama, Syria.  - **Sesame Oil**: from *Sesamum indicum* *L*. (Hama, Syria), supplied by **Mediterranean Harvest***®,* Hama, Syria. |
| **Extraction** | - **EVOO**: Cold-pressed, complying with IOOC Standard COI/T.15/NC No 3/Rev. 14 (2019) *****.  - **Sesame Oil**: Cold-pressed following ISO 11040:2022, Codex Alimentarius STAN 210-2019, and USP 43-NF 38 (2024). |
| **Quality Standards** | - **EVOO**: Free acidity ≤ 0.8%, peroxide value ≤ 15 meq/kg.  - **Sesame Oil**: Total phenolic content ≥ 0.5%, peroxide value ≤ 10 meq/kg. |
| **Preservation** | - Container: Dark glass bottles with nitrogen flushing.  - Storage: 4°C, away from light.  - Usage: Within 6 months (batches: February 2024).  - Sealing: Tightly sealed during testing to prevent oxidation/contamination. |
| **B. Plant Extracts** | |
| **Collection** | Psyllium seeds (*Plantago ovata*) and dandelion roots/leaves (*Taraxacum officinale*) were wild-harvested from the Damascus countryside (GPS coordinates: 33.51°N, 36.29°E) during April 2024. (Figure 2 A, B). |
| **Identification** | - **Botanical Authentication**: By Prof. Imad Alkadi (Botany Department, Faculty of Pharmacy, AIU, Damascus, Syria).  - **Voucher Specimens**: Deposited at Syrian National Herbarium (Plantago ovata: SNH-2024-005; Taraxacum officinale: SNH-2024-006). |
| **Drying** | The plants were dried in an oven at 40 °C until a consistent weight was achieved (approximately 72 hours). |
| **Grinding** | Each plant was ground separately using a milling machine (Retsch®, Hamburg, Germany). |
| **Extraction** | Forty grams of each plant powder were continuously extracted using a Soxhlet apparatus (Lab-Line Instruments®, Inc., Melrose Park, Illinois, USA) with a solvent mixture (cyclohexane: chloroform, 3:1 v/v) for 10 hours at 70 °C. |
| **Solvent Removal** | The solvent was eliminated using a rotary evaporator (Evolve®, Luqa, Malta, EU) at 40 °C. |
| **Final Product** | Lab-produced extracts (Psyllium seeds, Dandelion leaves/roots) dated 2024. |
| **Preservation** | Extracts were stored in airtight amber glass containers at 4°C, protected from light and humidity, and used within 6 months to ensure stability and prevent microbial or oxidative degradation. |
| **Ethical & Legal Compliance** | - **Permits**: Syrian Ministry of Agriculture (SY-AGR-2024-1358). - **Nagoya Protocol**: Adhered to for ABS compliance. - **No endangered species utilized.** |
| **EVOO:** Extra Virgin Olive Oil**; AIU:** Arab International University**; SNH:** Syrian National Herbarium**; ABS:** Access and Benefit-Sharing**; PV**: Peroxide Value; **meq/kg**: milliequivalents per kilogram.  *: Tiwari SW, Sah ANJIJPER. Effect of Apricot Fruit and Kernel Extracts on in-vitro Dissolution of Cholesterol Gallstones: Implication for Development of Potent Anti-cholilithiaticc agent. Indian Journal of Pharmaceutical Education and Research (Indian J Pharm Educ Res). 2020;54:755-60. | |
