# Supplementary material for: Synergistic effects of sesame oil, extra virgin olive oil, psyllium extract, and dandelion extract on cholesterol gallstone dissolution: An in vitro comparative study against Rowachol®
Source: PLoS One. 2025 Oct 14;20(10):e0334496. doi: 10.1371/journal.pone.0334496 (PMC12520339; doi:10.1371/journal.pone.0334496)
Supplement: S3 Table — (DOCX) [file pone.0334496.s003.docx]

| **Supplementary Table 3:** Chemical Composition and Gallstone-Dissolving Mechanisms of Plant Oils and Extracts | | | | | |
| --- | --- | --- | --- | --- | --- |
| **Plant Oils** | | | | | |
| **Component** | **KI** | | **Sesame Oil (%)** | **EVOO (%)** | **Role in Gallstone Dissolution *** |
| **Fatty Acids** | | | | | |
| Oleic Acid | 1600 | | 39.5 | 72.3 | Reduces cholesterol concentration in bile, improves bile flow. |
| Linoleic Acid | 1650 | | 44.2 | 9.8 | Anti-inflammatory, reduces cholesterol gallstone formation. |
| Palmitic Acid | 1580 | | 11.1 | 12.5 | supports liver health and reduces fat accumulation in bile. |
| **Phenolic Compounds** | | | | | |
| Hydroxytyrosol | 1180 | | ND | 5.2 | Strong antioxidant, reduces oxidative stress in the liver and gallbladder. |
| Oleuropein | 1950 | | ND | 3.8 | Stimulates bile secretion, reduces cholesterol concentration in bile. |
| Sesamin | 2100 | | 1.5 | ND | Anti-inflammatory, protects liver cells from damage. |
| Sesamolin | 2150 | | 0.9 | ND | Supports liver health, reduces gallstone formation. |
| **Vitamins & Antioxidants** | | | | | |
| Vitamin E | 1850 | | 0.3 | 1.2 | Protects liver and gallbladder cells from free radical damage. |
| Squalene | 1900 | | 0.1 | 0.4 | Antioxidant, supports liver health. |
| **Secondary Compounds** | | | | | |
| Chlorophyll | - | | TR | TR | Supports liver health, reduces oxidative stress. |
| Carotenoids | - | | ND | 0.2 | Antioxidant, protects liver and gallbladder cells. |
| **Total Identified** | | | **97.6** | **95.4** |  |
| **Plant Extracts** | | | | | |
| **Component** | | **Psyllium Extract (%)** | | **Dandelion Extract (%)** | **Role in Gallstone Dissolution *** |
| **Fibers & Other Compounds** | | | | | |
| Arabinoxylan | | 65.0 | | ND | Reduces cholesterol absorption, improves intestinal motility, lowers cholesterol in bile. |
| **Active Compounds** | | | | | |
| Taraxacin | | ND | | 12.3 | Stimulates bile secretion, reduces cholesterol concentration in bile. |
| Flavonoids | | ND | | 8.7 | Reduces inflammation, protects liver and gallbladder cells. |
| Polyphenols | | ND | | 5.0 | Reduces oxidative stress, prevents gallstone formation. |
| Bitter Compounds | | ND | | 3.0 | Stimulates bile secretion, improves digestion. |
| **Total Identified** | | **65.0** | | **29.0** |  |
| **KI**: Kovats Index for GC-MS identification; **EVOO**: Extra Virgin Olive Oil; **TR**: Trace amount (<0.05%).; **ND**: Not detected; *: Arrout A, El Ghallab Y, Yafout M, Lefriyekh MR, Said AAHJPP: Medicinal plants for gallstones: a cross-sectional survey of Moroccan patients. 2024, 4:100524. 10.1016/j.phyplu.2024.100524. | | | | | |
