# Supplementary material for: Synergistic effects of sesame oil, extra virgin olive oil, psyllium extract, and dandelion extract on cholesterol gallstone dissolution: An in vitro comparative study against Rowachol®
Source: PLoS One. 2025 Oct 14;20(10):e0334496. doi: 10.1371/journal.pone.0334496 (PMC12520339; doi:10.1371/journal.pone.0334496)
Supplement: S4 Table — (DOCX) [file pone.0334496.s004.docx]

| **Supplementary Table 4:** Extraction Protocol of 100 mg Terpenes from Rowachol® Capsules | | | |
| --- | --- | --- | --- |
| **Step** | **Description** | **Details/Quantity** | **Devices (Model/Manufacturer)** |
| **1. Capsule Calculation** | Determine the number of capsules needed to obtain 100 mg pure terpenes. | - Each capsule contains **67 mg terpenes**: - Pinene (17 mg) - Camphene (5 mg) - Cineol (2 mg) - Menthone (6 mg) - Menthol (32 mg) - Borneol (5 mg). - Capsules required: **2 capsules**. | - |
| **2. Terpene Extraction** | Separate terpenes from the carrier oil using an organic solvent. | - Solvent: **Ethanol** (10 mL). - **Ultrasonic Bath** for mixing. | Ultrasonic Bath (Branson 2800, USA). |
| **3. Layer Separation** | Separate the solvent (containing terpenes) from the oil. | - Centrifugation: **8000 rpm for 15 minutes**. - **Upper layer**: Solvent + Terpenes. | Centrifuge (Eppendorf 5430, Germany). |
| **4. Terpene Purification** | Remove the organic solvent to obtain pure terpenes. | Evaporation under vacuum at **40°C**. | Rotary Evaporator (Buchi R-300, Switzerland). |
| **HPLC**: High-Performance Liquid Chromatography; **rpm**: Revolutions per minute. | | | |
