# Supplementary material for: Synergistic effects of sesame oil, extra virgin olive oil, psyllium extract, and dandelion extract on cholesterol gallstone dissolution: An in vitro comparative study against Rowachol®
Source: PLoS One. 2025 Oct 14;20(10):e0334496. doi: 10.1371/journal.pone.0334496 (PMC12520339; doi:10.1371/journal.pone.0334496)
Supplement: S5 Table — (DOCX) [file pone.0334496.s005.docx]

| **Supplementary Table 5:** Specifications of BioIVT Bile Juice Used in the Study | | |
| --- | --- | --- |
| **Component** | **Specifications** | **Analytical Method** |
| **Source** | Human donors’ post-cholecystectomy (cholesterol stones). | - |
| **Bile Salts** | - Taurocholate: 45–55%  - Glycocholate: 30–40% | HPLC (Agilent 1260) |
| **Cholesterol** | 4–6 mM | HPLC with UV/Vis detector |
| **Phospholipids** | Lecithin: 1.5–2 mM | GC-MS (Shimadzu QP2010) |
| **pH** | 7.2–7.6 | pH meter (Mettler Toledo) |
| **Osmolality** | 290 ± 10 mOsm/kg | Osmometer (Wescor 5600) |
| **Preservatives** | 0.05% Sodium Azide | - |
| **Volume/Container** | 350 mL | - |
| **Storage** | -80°C in CryoBag® vacuum-sealed packaging | - |
| **Shelf Life** | 24 months | Manufacturer’s Certificate of Analysis (CoA) |
| HPLC: High-Performance Liquid Chromatography; GC-MS: Gas Chromatography-Mass Spectrometry; UV/Vis: Ultraviolet-Visible spectroscopy; CoA: Certificate of Analysis; mM: Millimolar; mOsm/kg: Milliosmoles per kilogram. | | |
