# Supplementary material for: Synergistic effects of sesame oil, extra virgin olive oil, psyllium extract, and dandelion extract on cholesterol gallstone dissolution: An in vitro comparative study against Rowachol®
Source: PLoS One. 2025 Oct 14;20(10):e0334496. doi: 10.1371/journal.pone.0334496 (PMC12520339; doi:10.1371/journal.pone.0334496)
Supplement: S6 Table — (DOCX) [file pone.0334496.s006.docx]

| **Supplementary Table 6:** Mixing and Dissolution Procedures for Groups | | | | | | |
| --- | --- | --- | --- | --- | --- | --- |
| **Group** | | **Code** | **Composition (v/v)** | **Final Concentration (mg/mL)** | **Purpose** | **Mixing Procedure** |
| **Experimental Groups** | **G1** | SO+BJ | 10% Sesame Oil + 90% Bile Juice | 100 mg/mL Sesame Oil + 900 mg/mL Bile Juice | Test sesame oil efficacy | Mix components using a vortex device (Vortex-Genie 2, Scientific Industries, USA) at 2000–3000 rpm for 30 seconds to 1 minute, then place the tube in a preheated water bath at 37°C for 5 minutes before adding the stone. |
|  | **G2** | EVOO+BJ | 10% Extra Virgin Olive Oil + 90% Bile Juice | 100 mg/mL EVOO + 900 mg/mL Bile Juice | Test olive oil efficacy |  |
|  | **G3** | SO+EVOO+BJ | 5% Sesame Oil + 5% EVOO + 90% Bile Juice | 50 mg/mL Sesame Oil + 50 mg/mL EVOO + 900 mg/mL Bile Juice | Test oil synergy |  |
|  | **G4** | PE+SO+EVOO+BJ | 5% Psyllium Extract + 5% SO/EVOO Mix + 90% Bile Juice | 50 mg/mL Psyllium Extract + 50 mg/mL SO/EVOO + 900 mg/mL Bile Juice | Test psyllium efficacy |  |
|  | **G5** | DE+SO+EVOO+BJ | 5% Dandelion Extract + 5% SO/EVOO Mix + 90% Bile Juice | 50 mg/mL Dandelion Extract + 50 mg/mL SO/EVOO + 900 mg/mL Bile Juice | Test dandelion efficacy |  |
|  | **G6** | DE+PE+SO+EVOO+BJ | 2.5% Dandelion Extract + 2.5% Psyllium Extract + 5% SO/EVOO Mix + 90% Bile Juice | 25 mg/mL Dandelion Extract + 25 mg/mL Psyllium Extract + 50 mg/mL SO/EVOO + 900 mg/mL Bile Juice | Test herbal synergy |  |
| **Control Group** | **G7** | Rowachol+BJ | 10% Rowachol® + 90% Bile Juice | 100 mg/mL Rowachol® + 900 mg/mL Bile Juice | Gold standard control | Add 0.1% Tween 80 as an emulsifier to prevent phase separation, then mix components using a vortex device (Vortex-Genie 2, Scientific Industries, USA) at 2000–3000 rpm for 30 seconds to 1 minute, and place the tube in a preheated water bath at 37°C for 5 minutes before adding the stone. |
| **SO**: Sesame Oil; **BJ**: Bile Juice; **EVOO**: Extra Virgin Olive Oil; **PE**: Psyllium Extract; **DE**: Dandelion Extract; **SO/EVOO** **Mix**: A 1:1 mixture of Sesame Oil and Extra Virgin Olive Oil; **Rowachol®:** A commercial drug containing terpenes (menthol, pinene, etc.) used for gallstone dissolution; **Tween 80**: A non-ionic surfactant used as an emulsifier to prevent phase separation; **rpm**: Revolutions per minute (speed of vortex mixing**); mg/mL**: Milligrams per milliliter (concentration unit); **v/v**: Volume per volume (ratio of components in the mixture). | | | | | | |

|  | | | |
| --- | --- | --- | --- |
| **Group** | **Composition**  **(Active material (100mg/ml) in BJ)** | **Mixing Protocol for each tube** | |
| G1_(SO)_ | Active: Sesame Oil | 1. Add **1.087 mL SO** (calculated for 1000 mg using density = 0.92 g/mL). 2. Top up to **10 mL with bile juice**. | Mix components using a vortex device (Vortex-Genie 2, Scientific Industries, USA) at 2000–3000 rpm for 30 seconds to 1 minute, then place the tube in a preheated water bath at 37°C for 5 minutes before adding the stone. |
| G2_(EVOO)_ | Active: Extra Virgin Olive Oil | 1. Add **1.099 mL EVOO** (calculated for 1000 mg using density = 0.91 g/mL). 2. Top up to **10 mL with bile juice**. |  |
| G3_(SO+EVOO)_ | Active: 50% Sesame Oil + 50% EVOO | 1. Add **0.543 mL SO** (500 mg) + **0.549 mL EVOO** (500 mg). 2. Top up to **10 mL with bile juice**. |  |
| G4_(PE+SO+EVOO)_ | Active: 50% Psyllium + 50% oils (25% SO + 25% EVOO) | 1. Weigh **500 mg Psyllium**. 2. Add **0.272 mL SO** (250 mg) + **0.275 mL EVOO** (250 mg). 3. Top up to 10 mL with bile juice. |  |
| G5_(DE+SO+EVOO)_ | Active: 50% Dandelion + 50% oils (25% SO + 25% EVOO) | 1. Weigh **500 mg Dandelion**. 2. Add **0.272 mL SO** + **0.275 mL EVOO**. 3. Top up to 10 mL with bile juice. |  |
| G6_(DE+PE+SO+EVOO)_ | Active: 25% Dandelion + 25% Psyllium + 50% oils (25% SO + 25% EVOO) | 1. Weigh **250 mg Dandelion** + **250 mg Psyllium**. 2. Add **0.272 mL SO** + **0.275 mL EVOO**. 3. Top up to 10 mL with bile juice. |  |
| G7_(Rowachol)_ | Active: Rowachol® | 1. Add **1.111 mL Rowachol®** (calculated for 1000 mg using density ≈ 0.9 g/mL). 2. Top up to **10 mL with bile juice**. | Add 0.1% Tween 80 as an emulsifier to prevent phase separation, then mix components using a vortex device (Vortex-Genie 2, Scientific Industries, USA) at 2000–3000 rpm for 30 seconds to 1 minute, and place the tube in a preheated water bath at 37°C for 5 minutes before adding the stone. |
|  | | | |
